# AGING TRAJECTORY OF THE JAPAN SCIENCE AND TECHNOLOGY AGENCY INDEX OF COMPETENCE (JST-IC): THE OTASSHA STUDY

**DOI:** 10.1093/geroni/igae098.2600

**Published:** 2024-12-31

**Authors:** Hisashi Kawai, Keigo Imamura, Manami Ejiri, Hiroyuki Sasai, Yoshinori Fujiwara, Kazushige Ihara, Hirohiko Hirano, Shuichi Obuchi

**Affiliations:** Tokyo Metropolitan Institute for Geriatrics and Gerontology, Tokyo, Japan; Tokyo Metropolitan Institute for Geriatrics and Gerontology, Tokyo, Japan; Tokyo Metropolitan Institute for Geriatrics and Gerontology, Tokyo, Japan; Tokyo Metropolitan Institute for Geriatrics and Gerontology, Tokyo, Japan; Tokyo Metropolitan Institute for Geriatrics and Gerontology, Tokyo, Japan; Hirosaki University, Hirosaki, Aomori, Japan; Tokyo Metropolitan Institute for Geriatrics and Gerontology, Tokyo Metropolitan Institute for Geriatrics and Gerontology, Tokyo, Japan; Tokyo Metropolitan Institute for Geriatrics and Gerontology, Tokyo, Japan

## Abstract

The Japan Science and Technology Agency Index of Competence (JST-IC) was developed to measure higher levels of competence than measured by the Tokyo Metropolitan Institute of Gerontology Index of Competence (TMIG-IC), which evaluates higher-level functional capacity necessary for independent daily life. This study compared the aging trajectory between the JST-IC and TMIG-IC among community-dwelling older Japanese individuals. The participants were 775 adults who participated in the health check-up of the Otassha study three or more times between 2014 and 2019. Three patterns of aging trajectories of the JST-IC and TMIG-IC were identified using group-based trajectory modeling. Each subscale trajectory of the JST-IC included technology usage, information practices, life management, and social engagement. Scores for the high (30.4%), medium (15.5%), and low (15.2%) JST-IC trajectories decreased by 1-2 points according to age. High stability (49.9%), late-onset decrease (41.2%), and early onset decrease (8.9%) TMIG-IC trajectories were identified, and the late-onset and early onset decrease groups decreased by 1-3 points with age. Scores for technology usage, life management, and social engagement declined with age, similar to the total score of the JST-IC; however, scores for information practice tended to remain at the same level as at 65 years of age. The participants in this study participated in a longitudinal survey, and higher-level functional capacity was almost maintained; however, we found that the JST-IC score gradually declined with age in all patterns. It has been suggested that this decline is associated with a decline in technology usage, life management, and social engagement.

